# Mitochondrial-Directed Antioxidant Reduces Microglial-Induced Inflammation in Murine In Vitro Model of TC-83 Infection

**DOI:** 10.3390/v10110606

**Published:** 2018-11-02

**Authors:** Forrest Keck, Daud Khan, Brian Roberts, Nitin Agrawal, Nishank Bhalla, Aarthi Narayanan

**Affiliations:** 1 National Center for Biodefense and Infectious Diseases, George Mason University, Manassas, VA 20110, USA; fkeck@masonlive.gmu.edu (F.K.); nbhalla@gmu.edu (N.B.); 2 Krasnow Institute for Advanced Study, George Mason University, Fairfax, VA 22030, USA; mkhan51@masonlive.gmu.edu (D.K.); nagrawa2@gmu.edu (N.A.); 3 Leidos Health Sciences, 5202 Presidents Court, Suite 110, Frederick, MD 21704, USA; BRIAN.A.ROBERTS@leidos.com

**Keywords:** Venezuelan equine encephalitis virus, microglia, inflammation, mitochondrial dysfunction

## Abstract

Venezuelan equine encephalitis virus (VEEV) is an arbovirus that is associated with robust inflammation that contributes to neurodegenerative phenotypes. In addition to triggering central nervous system (CNS) inflammation, VEEV will also induce mitochondrial dysfunction, resulting in increased cellular apoptosis. In this study, we utilize the TC-83 strain of VEEV to determine the role of mitochondrial oxidative stress in mediating inflammation elicited by murine brain microglial cells. Using an in vitro model, we show that murine microglia are susceptible to TC-83 infection, and that these cells undergo mitochondrial stress as the result of infection. We also indicate that bystander microglia contribute more significantly to the overall inflammatory load than directly infected microglia. Use of a mitochondrial targeted antioxidant, mitoquinone mesylate, greatly reduced the pro-inflammatory cytokines released by both direct infected and bystander microglia. Our data suggest that release of interleukin-1β, a key instigator of neuroinflammation during VEEV infection, may be the direct result of accumulating mitochondrial stress. This data improves our understanding inflammation elicited by murine microglia and will aid in the development of more accurate in vitro and in vivo murine model of VEEV-induced neuroinflammation.

## 1. Introduction

Neurotropic Venezuelan equine encephalitis virus (VEEV) causes outbreaks of febrile and neurological disease in equines and humans. Transmitted by mosquitos, epidemic strains of VEEV are known to circulate from Texas to Peru and periodically cause outbreaks in countries with enzootic circulation [1]. Highly infectious via the aerosol route, VEEV is classified as an emerging infectious pathogen while having previously been used to create bioweapons [2,3]. While there are no licensed vaccines available for public use, the live-attenuated TC-83 vaccine is available for at-risk personnel. VEEV is a positive-sense, single-stranded RNA virus belonging to the genus *Alphavirus*, in the family *Togaviridae* [4,5]. It is well accepted that VEEV enters the central nervous system (CNS) through the olfactory tract and causes encephalitis characterized by neuronal death and inflammation [6,7,8]. In addition to the direct effects of VEEV infection, the virus has been observed to cause secondary neuronal damage independent of direct viral events [7,9,10,11,12]. The secondary neuronal damage has been linked to several pro-inflammatory cytokines induced during VEEV infection and controlled by glycogen synthase kinase-3β, including interferon-γ (IFNγ), interleukin-1β (IL-1β), IL-6, IL-8, and IL-12 [11,13,14,15,16]. We hypothesize that these inflammatory mediators are produced as the result of activated bystander microglia.

Microglia are the resident CNS macrophages and therefore play a significant role in the progression of neurological diseases as the result of their immunomodulatory role [17,18,19,20]. Microglia are known to rapidly induce several pro-inflammatory cytokines, including members of the IL-1 family. In microglia, many IL-1 family cytokines exist as preformed precursors that can undergo instant cytokine maturation and release upon stimulation [21,22]. Of these, IL-1β is often implicated as the main instigator of a pro-inflammatory state, as it is the major soluble form of IL-1 and has numerous other targets, including T-cells, B-cells, monocytes, and macrophages [23]. Microglial morphology, while often changed during activation, is not an infallible indicator of cytokine production, as an amplified cytokine response requires the initial release of TNFα and its subsequent autocrine signaling cascade [22]. Upon activation, microglia will express integrin αM/β2 (also called cluster of differentiation molecule 11b or CD11b), which mediates a number of immune responses, including adhesion, migration, and phagocytosis [24].

In a number of neurodegenerative diseases and viral infections, neuroinflammation has been linked to abrogation of normal mitochondrial function [25,26,27,28]. These alterations include altered redox status, dysregulated energy metabolism, and structural and functional changes to key respiratory chain complexes and enzymes [25,26,27,28,29,30,31]. We have previously reported that the TC-83 strain of VEEV can induce mitochondrial dysfunction that includes structural alterations, increased accumulation of reactive oxygen species (ROS), loss of the mitochondrial membrane potential (MMP), induction of mitochondrial fission, and increased Parkin-mediated mitophagy [32]. We have also indicated that mitochondrial damage and the inflammatory response are interconnected in a manner that contributes to the establishment of a successful infection [33].

Much of this research has been investigated in the context of one-dimensional cell culture models that do not capture the complexity of the neuronal microenvironment. Several murine models of VEEV infection have begun to characterize long-term viral-induced inflammation in the brain [7,13,34,35,36]. These models confirm the cytokine induction described in cell culture models of infection, and have even implicated the pro-inflammatory environment in increased leukocyte extravasation that results in further increased neuroinflammation [34]. However, these murine models have not studied mitochondrial function in the context of VEEV infection, and much remains to be elucidated about the murine neuroinflammatory environment. This study investigates how an in vitro murine model of VEEV infection can be used to study neuroinflammation and viral interactions with the blood brain barrier using a well-established murine microglial cell line.

In this study, we utilize the TC-83 strain of VEEV to illustrate that murine microglia are susceptible to infection and that infection results in mitochondrial dysfunction. We determine that infected murine microglia produce several pro-inflammatory cytokines as the result of direct infection, including IFNγ, IL-1α, IL-1β, IL-6, and IL-12. Bystander murine microglia contribute more greatly to the overall inflammatory environment than directly infected microglia, with 100+ fold increases in the pro-inflammatory cytokines IL-1β, IL-6, and IL-8. We show that these direct and indirect inflammatory events are the result of mitochondrial dysfunction, which can be abrogated using a mitochondrially targeted antioxidant. This data reveals a novel connection between mitochondrial dysfunction the ensuing neuroinflammatory response, improving our understanding of VEEV-induced inflammation in both in vitro and in vivo murine models of infection.

## 2. Materials and Methods

### 2.1. Viruses and Cell Lines

U-87 MG human astrocytoma cells (American Type Culture Collection; HTB-14), VERO African green monkey kidney cells (American Type Culture Collection; CCL-81), and BV2 murine microglial cells (Duke University, Brian Hawkins) were cultured in Dulbecco’s modified Eagle’s medium (DMEM) (Quality Biological, Gaithersburg, MD, USA) with 10% fetal bovine serum (FBS) (Gibco, Gaithersburg, MD, USA). Live-attenuated VEEV (TC-83 strain) was obtained from BEI Resources (Manassas, VA, USA).

### 2.2. Infection and Plaque Assay

Cells were seeded at a density of 10^4^ cells per 100 μL in clear 96-well F-bottom plates overnight, followed by the addition of DMEM with TC-83 at an MOI of 2. The cells were incubated for 1 h at 37.1 °C with viral overlay. The viral inoculum was then removed, cells were washed once with DMEM, and media replaced prior to subsequent incubation. The viral supernatants were quantitated using plaque assay for determination of extracellular viral titers. For the plaque assay, VERO cells were plated in 12-well F-bottom plates at a density of 2 × 10^5^ cells/well and cultured in DMEM at 37.1 °C, 5% CO_2_. Serially diluted virus solution was added to plates for 1 h, after which wells were covered with 2× Eagle’s minimum essential medium (EMEM) for plaques (Quality Biological, USA) containing 0.6% agarose (Life Technologies, Carlsbad, CA, USA). Two days post-infection, the cells were fixed with 10% formaldehyde for 1 h. The fixative and agarose plugs were then removed, and cells stained with a crystal violet solution containing 1% crystal violet and 20% ethanol. Crystal violet was rinsed off the plates using distilled water and plaques were subsequently counted.

### 2.3. Inhibitors and Cell Viability

The mitochondrially targeted antioxidant mitoquinone mesylate (MedKoo Biosciences, Morrisville, NC, USA) and the NF-κB inhibitor BAY 11-7082 (Selleckchem, Houston, TX, USA) were investigated in the context of VEEV infection. The non-toxic ranges of our therapeutics were determined in BV2 and U-87 MG cells using the CellTiter Glo assay (Promega, Madison, WI, USA) according to the manufacturer’s instructions.

### 2.4. Quantitative Real-Time Polymerase Chain Reaction

Cells were lysed, and viral RNA extracted using the QIAamp Viral RNA kit (Qiagen, Gaithersburg, MD, USA) according to the manufacturer’s instructions. The viral RNA was then quantitated using qRT-PCR with primers and probe targeting the capsid sequence of VEEV TC-83 [14]. Pre-cycling conditions were in accordance with manufacturer’s instructions for the Verso 1-step RT-qPCR kit (ThermoFisher, Waltham, MA, USA). An annealing temperature of 61 °C was used for the 40 amplification cycles. The sequences are as follows: forward primer TCTGACAAGACGTTCCCAATCA, reverse primer GAATAACTTCCCTCCGACCACA, and probe 6-FAM/TGTTGGAAG/ZEN/GGAAGATAAACGGCTACGC/IABkFQ (Integrated DNA Technologies, Coralville, IA, USA). Absolute quantification was calculated based on the threshold cycle (*C*t) relative to the standard curve.

### 2.5. Caspase Quantitation

Induction of caspase-3/7 was measured using the Caspase-Glo 3/7 Assay (Promega, USA) according to the manufacturer’s instructions. Samples were read using a Beckman Coulter DTX 880 multimode plate reader.

### 2.6. Cellular Reactive Oxygen Species

The production of reactive oxygen by cells was measured using a fluorogenic dye (2′,7′-dichlorofluorescin diacetate; DCF) assay according to the manufacturer’s instructions (Abcam, Cambridge, MA, USA). Fluorescence was measured using a Beckman Coulter DTX 880 fitted with a 485/535 Ex/Em filter. As a positive control, cells were treated with 100 μM tert-butyl hydrogen peroxide (TBHP) to induce DCF fluorescence.

### 2.7. Mitochondrial Membrane Potential

Maintenance of the mitochondrial membrane potential (MMP) was determined using the cell permeant, positively-charged, red-orange dye tetramethylrhodamine, ethyl ester (TMRE) (Abcam, USA). Fluorescence was measured using a Beckman Coulter DTX 880 fitted with a 549/575 Ex/Em filter. As a positive control, cells were treated with 50 μM carbonyl cyanide 4-(trifluoromethoxy) phenylhydrazone (FCCP), which is known to disrupt MMP.

### 2.8. Live Cell Imaging

The time-lapse phase contrast imaging of murine microglial cell activation was monitored using the EVOS FL Auto cell imaging system (ThermoFisher, USA). We utilized the EVOS to obtain a time-lapse sequence of digital images taken every 6 min for 14 h total. All images were obtained with the 10× objective. Cell counts for microglia in various stages of activation were determined using ImageJ software (Version 1.52e/11, NIH, Bethesda, MD, USA).

### 2.9. Direct Flow Cytometry

Harvested cells were washed with medium prior to cell suspension adjustment to reach a final concentration of 5 × 10^6^ cells/mL in ice cold phosphate buffered saline (PBS) with 10% fetal calf serum (FCS) and 1% sodium azide. Cells were stained with 1µg/mL of anti-CD11b antibody labeled with phycoerythrin-cyanine-5 (Abcam, USA) and incubated at 4 °C for 1 h. Cells were then washed three times by centrifugation at 400 *g* for 5 min and resuspended in 500 µL of ice cold PBS, 10% FCS, and 1% sodium azide. To preserve the surface antigen presentation, cells were fixed with 1% paraformaldehyde in PBS for 12 min. Cells were subsequently washed three times by centrifugation as previously described. Fixed cells were stored at 4 °C until processed using a BD FACSAria II (BD Biosciences, San Jose, CA, USA). Data were analyzed using BD FACSDiva Software Version 8.0.

### 2.10. Confocal Microscopy

BV2 microglia were first fixed in 4% paraformaldehyde for 10 min. Cells were then permeabilized with 0.1% Triton X-100 in PBS for 10 min prior to blocking with 1% bovine serum albumin (BSA) in 0.3 M glycine with 0.01% Triton X100 in PBS for 30 min at room temperature. The cells were stained with mouse monoclonal antibody to TOMM20 (Abcam, USA) and polyclonal goat serum to TC-83 capsid protein (BEI Resources, Manassas, VA, USA) at room temperature for 1 h. Alexa Fluor 488-conjugated goat anti-mouse (Invitrogen, Carlsbad, CA, USA) and Alexa Fluor 568-conjugated donkey anti-goat (Invitrogen, USA) secondary antibodies were added for 1 h at room temperature in the dark. The cells were mounted using Fluoromount G with DAPI (Southern BioTech, Birmingham, AL, USA) and visualized using the Nikon TE2000U confocal microscope. All confocal images were processed suing NIS Elements software (Nikon, Melville, NY, USA).

### 2.11. Inflammatory Cytokine Array

To detect changes in the cytokines being released by VEEV infected cells, samples were processed using the human 10-plex Ciraplex array (Aushon, Billerica, MA, USA) according to the manufacturer’s instructions. The Aushon Cirascan ASP-2010 was used to image the immunoassay. Cirascan images were analyzed using the Cirasoft Analysis software according to the 101-3FF-1-AB kit alignment. Analyte concentrations were converted from pg/mL to fold change versus time matched mock controls using Prism 5 software (GraphPad, LaJolla, CA, USA).

### 2.12. Statistical Analysis

The software Prism 5 (GraphPad, USA) was used for all statistical analyses. Data were presented as mean ± standard deviation. When appropriate, the number of replicates depicted by each graph is indicated in the figure legend. Results were analyzed by unpaired, two-tailed *t*-test. Differences were considered statistically different at *p* < 0.05.

## 3. Results

### 3.1. Murine Microglia are Susceptible to VEEV Infection

**Figure 1:**
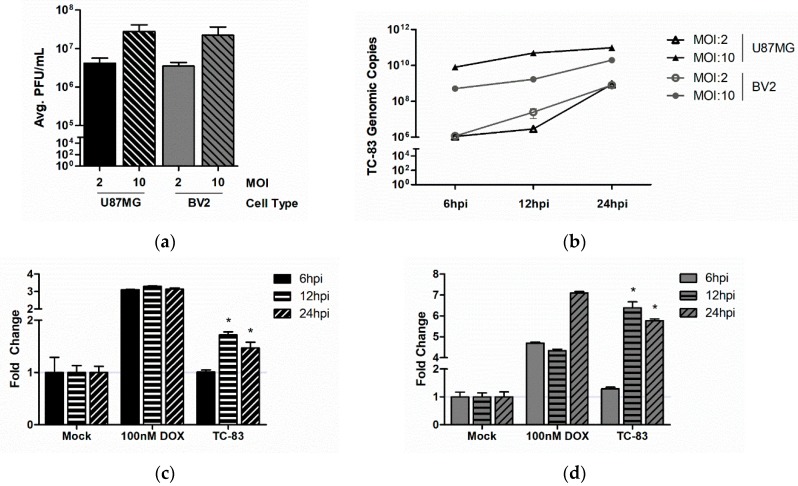
Murine microglia sustain viral loads and induce caspase in a manner similar to the accepted astrocyte model of TC-83 infection. U-87 MG astrocytes (black) and BV2 microglia (grey) were inoculated with TC-83 (MOI:2 or MOI:10). (**a**) Production of infectious virus was confirmed in all cells via plaque assay at 24 h post infection (hpi); (**b**) qRT-PCR of intracellular TC-83 genomic copies indicate successful viral replication in both cell lines. Activation of caspase-3/-7 was confirmed in (**c**) U-87 MG astrocytes and (**d**) BV2 microglia infected with TC-83 (MOI:2). Doxorubicin (DOX) was used as a positive control for caspase induction. The quantitative data are depicted as the means of three biologically independent experiments ± SD. * indicates significant difference (*p* < 0.05) between the indicated sample and the time-matched, mock control.

Establishment of productive VEEV infection in the brain is centered on the susceptibility of astrocytes and neurons to infection. Previous research involving VEEV-induced neuroinflammation led us to hypothesize that microglial cells also play an important role in the development of encephalitis. To this end, we first determined to what extent murine microglia (BV2) can be infected by TC-83. Plaque analysis revealed that murine microglia produced an equivalent number of infectious viral particles as the accepted human astrocytoma cell line (U-87 MG) (Figure 1a). The production of infectious virus mirrored a time- and dose-dependent increase in TC-83 genomic copies as measured by intracellular qRT-PCR (Figure 1b). Previous studies have indicated that TC-83 induces the caspase response in a mitochondrial-dependent manner, ultimately contributing to cell death during infection [32]. Here we demonstrate that BV2 microglia activate the caspase-3/-7 response to a greater extent than the U-87 MG astrocyte model (Figure 1c,d). These results indicate that TC-83 can infect murine microglia in vitro and produce cellular outcomes that mirror the accepted astrocytoma model.

### 3.2. TC-83 Infection Induces Mitochondrial Dysfunction in Murine Microglia

**Figure 2:**
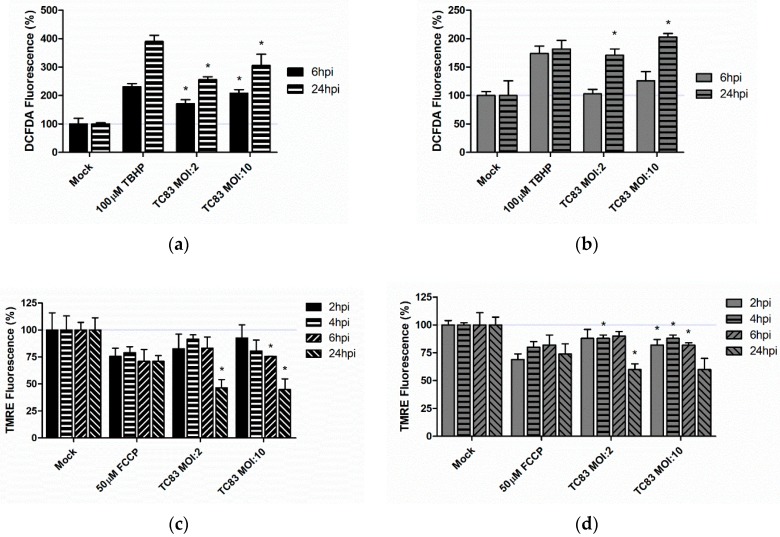
TC-83 infection induces mitochondrial dysfunction. U-87 MG astrocytes and BV2 microglia were inoculated with TC-83 (MOI:2 or MOI:10). Fluorescence of 2′,7′-dichlorofluorescin diacetate (DCFDA) was measured as an indicator of reactive oxygen species (ROS) accumulation in (**a**) U-87 MG and (**b**) BV2 cells. Tert-butyl hydrogen peroxide (TBHP) was used as a positive control for ROS accumulation. Fluorescence of tetramethylrhodamine ethyl ester (TMRE) was measured as an indicator of mitochondrial membrane potential (MMP) in TC-83 infected (**c**) U-87 MG and (**d**) BV2 cells. Carbonyl cyanide 4-(trifluoromethoxy) phenylhydrazone (FCCP) was used as a positive control for the abrogation of MMP. The quantitative data are depicted as the means of six biologically independent experiments ± SD. * indicates significant difference (*p* < 0.05) between the indicated sample and the time-matched mock control.

Accumulation of reactive oxygen species (ROS) and disruption of the mitochondrial membrane potential (MMP) are accepted markers of mitochondrial stress [25,26,32,33,37]. Our previous studies indicate that TC-83 infection causes a decrease in MMP and an increase in ROS in a manner that directly contributes to viral-induced cell death [32,33]. Here, we compare the extent of mitochondrial stress induced by TC-83 infection of murine microglia to the accepted human astrocytoma model. The murine microglia and human astrocytes were inoculated with TC-83 at either an MOI of 2 or 10, and ROS and MMP measured at biologically relevant time points. At 24 h post infection (hpi) with an MOI of 2, the ROS burden increased by 256% and 171% in U-87 MG and BV2 cell lines, respectively (Figure 2a,b). This increase in ROS corresponded with a decrease MMP in both cell lines. At 24 hpi, TC-83 infected astrocytes were displaying a 53.6% decrease in MMP compared to the uninfected control (Figure 2c). Correlating with the lesser ROS burden, BV2 microglia only experienced a 40% decrease in MMP at 24 hpi (Figure 2d). The accumulation of ROS and decrease in MMP was not MOI-dependent, and thus all subsequent experiments will be conducted using the MOI of 2. These results indicate that murine microglia experience a loss of mitochondrial function during TC-83 infection.

### 3.3. Microglial Activation as the Result of TC-83 Infection

**Figure 3:**
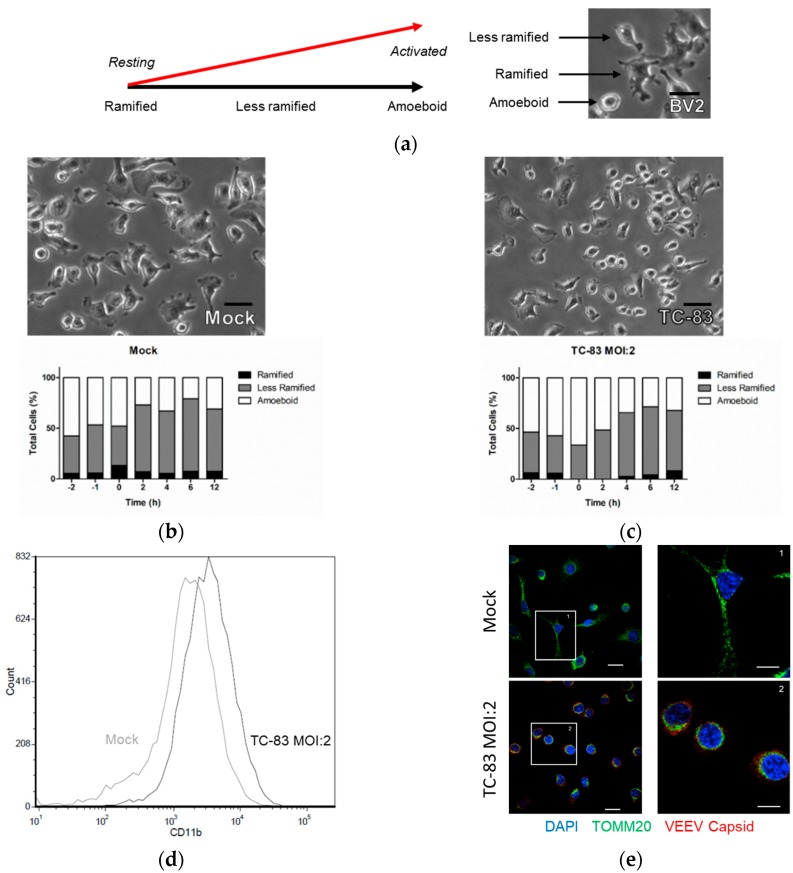
BV2 microglia become activated as the result of TC-83 infection. (**a**) Representative diagram of microglial morphology corresponding to activation and an example of these morphologies in BV2 cells. Live cell imaging of (**b**) mock uninfected and (**c**) TC-83 (MOI:2) infected BV2 microglia revealed cell morphologies during infection. ImageJ software was used to quantify the percentage of cells in each frame that were ramified, less ramified, or amoeboid. Scale bars for (**a**–**c**) represent 20 µm. (**d**) At 2 hpi, mock uninfected and TC-83 infected microglia were fixed with paraformaldehyde and incubated with PE-Cy5 conjugated CD11b antibody prior to direct flow cytometry analysis. See Appendix A, Figure A1 for gating strategy. (**e**) Confocal analysis of BV2 microglia fixed with paraformaldehyde at 2 hpi. Cells were stained with primary antibodies specific to TOMM20 mitochondrial protein and VEEV capsid prior to conjugation with Alexa Fluor secondary antibodies. Scale bars represent 10 μm. The quantitative data are depicted as the means of three biologically independent experiments.

Viral insult has previously been shown to activate microglia cells and play a critical role in the recruitment of innate immune responses [38,39,40,41,42]. Activated microglia undergo significant morphology alterations, transitioning from a ramified resting state to an amoeboid activated state (Figure 3a). We observed murine microglia in the context of TC-83 infection using live cell imaging to track cultures for 2 h pre- and 12 h post-viral inoculation. During this time course, the uninfected mock control had an average 8% resting, ramified microglia (Figure 3b). The number of cells displaying an activated phenotype decreased 27.3% by 12 h as cells settled and transitioned to a less activated state. Microglia that were inoculated with TC-83 instantly displayed a shift in morphology, with all cells in some stage of activation up to 2 h post inoculation, and up to 66.2% of cells in the fully activated state (Figure 3c). These phenotype changes were corroborated by flow cytometry analysis which indicates that TC-83 infected microglia express more of the CD11b activation marker than the uninfected control at 2 hpi (Figure 3d). The shift in morphology also corresponds with a retraction of the mitochondrial network, as indicated by the perinuclear accumulation of the trans-outer mitochondrial membrane protein TOMM20 in the TC-83 infected cells (compare Figure 3e(1,2)). These results indicate that the microglial cell lines become activated following TC-83 infection and undergo pronounced changes in morphology.

### 3.4. Microglial-Mediated Inflammatory Response to TC-83 Infection

#### 3.4.1. Direct TC-83 Infection of Microglia Produces Altered Inflammatory Cytokine Profile

**Figure 4:**
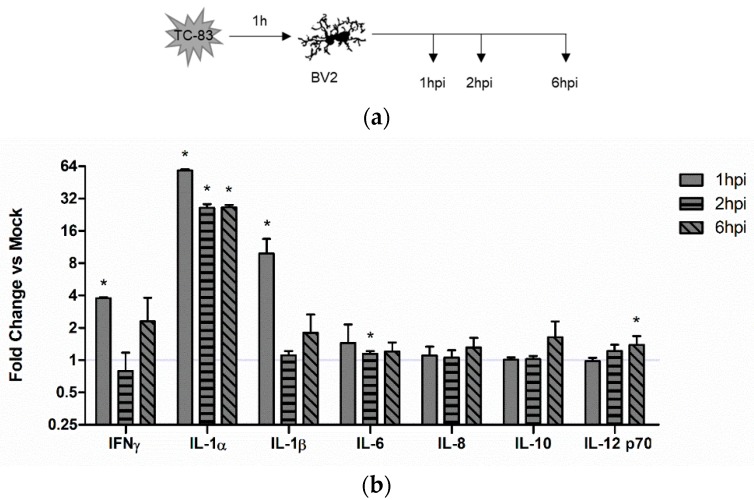
TC-83 induced inflammation in BV2 microglia. (**a**) Experimental workflow for the infection and collection of supernatants from BV2 cells. (**b**) Inflammatory cytokine profiles for mock uninfected and TC-83 (MOI:2) infected BV2 cells were measured with an Aushon Ciraplex kit. Cytokines are depicted as fold induction as compared to uninfected time-matched controls. The quantitative data are depicted as the means of three biologically independent experiments ± SD. * indicates significant difference (*p* < 0.05) between the indicated sample and the time-matched mock control.

We hypothesized that the activated BV2 microglia would produce inflammatory cytokines in a manner that correlates with the activation profile described in Figure 3c. Using the infection scheme described in Figure 4a, we analyzed BV2 microglia for cytokine production following direct infection with TC-83 (MOI:2). Using a 10plex cytokine array, we observed that TC-83 infected microglia experience a marked increase in IFN-γ, IL-1α, and IL-1β at 1 hpi as compared with the uninfected mock control (Figure 4b).

#### 3.4.2. Indirect Inflammatory Response Significantly Contributes to Overall Inflammatory Burden

**Figure 5:**
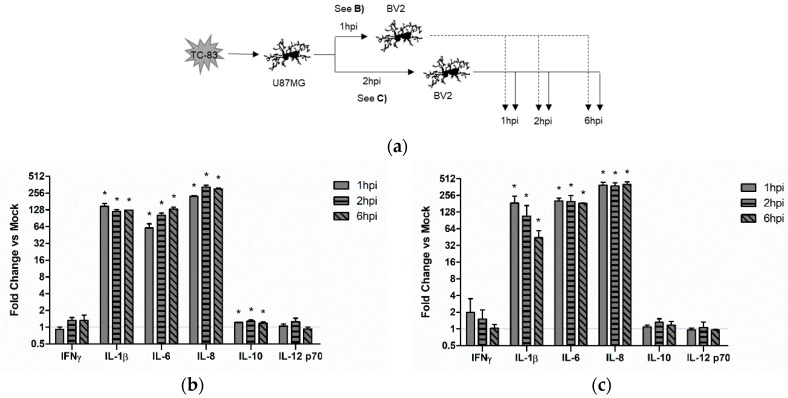
Bystander microglia produce more pro-inflammatory cytokines than direct infected microglia. (**a**) Experimental workflow indicating that U-87 MG astrocytes were infected with TC-83 (MOI:2) and supernatants removed at 1 and 2 hpi to be overlaid on naïve BV2 microglia. Inflammatory cytokine profiles for the (**b**) 1 hpi overlay and (**c**) 2 hpi overlay were measured with an Aushon Ciraplex kit. Cytokines are depicted as fold induction as compared to uninfected time-matched controls. The quantitative data are depicted as the means of three biologically independent experiments ± SD. * indicates significant difference (*p* < 0.05) between the indicated sample and the time-matched mock control.

While it is important to elucidate the inflammatory cytokines produced by directly infected microglia cells, we hypothesize that these microglia will not contribute as greatly to the overall inflammatory burden given the ability of VEEV capsid protein to inhibit host transcriptional machinery [43]. Instead, we hypothesize that bystander microglia will contribute more greatly to the overall inflammatory burden. To this end, we infected U-87 MG astrocytes with TC-83 (MOI:2) and removed supernatants from these infected cells at 1 and 2 hpi (Figure 5a). Supernatants were treated with anti-VEEV neutralizing Ab to prevent virions from infecting naïve BV2 cells. Furthermore, supernatants were tested by plaque assay to demonstrate lack of infecting virions before addition to naïve BV2 cells. These supernatants were then overlaid on naïve BV2 microglia, and inflammatory cytokines measured at 1, 2, and 6 h post overlay. We observed a significant increase in IL-1β, IL-6, and IL-8 produced by BV2 microglia when exposed to either 1hpi (Figure 5b) or 2hpi supernatants (Figure 5c) as compared with the uninfected mock control. IFN-γ was increased in both instances, but not significantly. The anti-inflammatory IL-10 was significantly induced in 1hpi supernatants, while IL-12 p70 was not significantly altered in either overlay. These results indicate that murine microglia can be activated by cytokines produced from TC-83 infected astrocytes in a manner that will contribute to the overall inflammatory load.

### 3.5. Therapeutic Intervention Rescues Mitochondrial Function in TC-83 Infected Microglia

**Figure 6:**
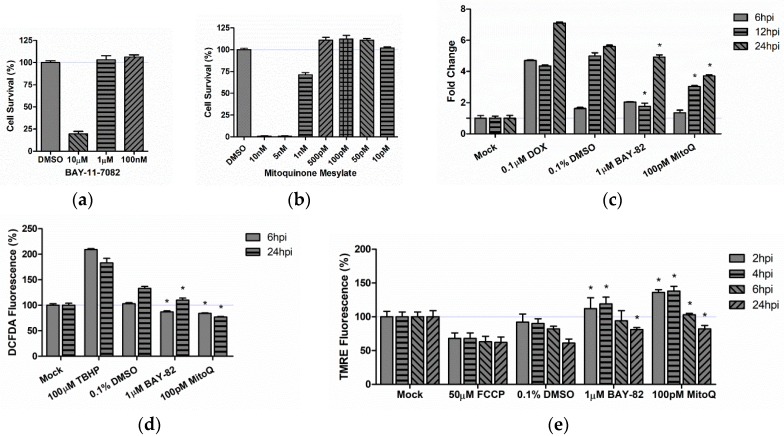
Mitochondrially directed therapeutics are effective at decreasing mitochondrial damage induced by TC-83 infection. Non-toxic ranges of (**a**) BAY 11-7082 (BAY-82) and (**b**) mitoquinone mesylate (MitoQ) were determined in BV2 microglia at 24 h post treatment. DMSO treated cells were maintained as a positive control for cell survival. BV2 microglia were treated with BAY-82, MitoQ, or the DMSO solvent control for 2 h prior to infection with TC-83 (MOI:2). Treated cells were analyzed for (**c**) caspase-3/-7 induction, (**d**) reactive oxygen species accumulation, and (**e**) maintenance of mitochondrial membrane potential using previously described methods. The quantitative data are depicted as the means of three biologically independent experiments ± SD. * indicates significant difference (*p* < 0.05) between the indicated sample and the time-matched DMSO control.

We hypothesized that the induction of inflammatory cytokines would be related to the mitochondrial dysfunction described in Figure 2. To determine the role of mitochondrial stress in the overall inflammatory profile, we utilized the mitochondrially targeted antioxidant mitoquinone mesylate (MitoQ). As a positive control, we utilized the anti-inflammatory molecule BAY 11-7082 (BAY-82), which was previously characterized by our group as a potent inhibitor of TC-83 [44]. We determined that the non-toxic dose for BAY-82 was 1 µM in BV2 (Figure 6a) and U-87 MG cells, and the non-toxic dose for MitoQ was 500 pM in BV2 cells (Figure 6b) and 100 pM in U-87 MG cells. All subsequent experiments were conducted using either 1 µM BAY-82, or 100 pM MitoQ.

Both primate and cell culture models of VEEV infection have indicated that the caspase response is activated as the result of infection [32,33,45]. This activation, partially mediated by mitochondrial dysfunction, drives a portion of viral-mediated apoptosis [32]. Therefore, we utilized the mitochondrial antioxidant, MitoQ, to determine whether reducing oxidative stress can decrease the extent of caspase activation noted in TC-83 infected murine microglia. We pre-treated BV2 microglia with BAY-82, MitoQ, or the solvent control (DMSO) prior to infection with TC-83 (MOI:2). We also included a mock uninfected control and doxorubicin (DOX) treated cells as a positive control for caspase-3/-7 induction. TC-83 infected, solvent treated (DMSO) microglia displayed a 6-fold induction of caspase-3/-7 as compared to the uninfected mock at 24 hpi (Figure 6c). When compared to the solvent control, the anti-inflammatory BAY-82 had a 320% decrease in caspase at 12 hpi, which decreased to a 70% decrease at 24 hpi. MitoQ had consistently lower caspase induction when compared to the solvent control, with 35%, 190%, and 210% reductions at 6, 12, and 24 hpi, respectively.

To determine if this reduction in caspase activation was the result of increased mitochondrial function, we queried BV2 microglia that had been treated with either BAY-82 or MitoQ for reduction of ROS burden and maintenance of MMP (Figure 6d). Our results indicate that the anti-inflammatory BAY-82 decreased overall ROS burden by 16% at 6 hpi and 22% at 24 hpi when compared to the TC-83 infected solvent control. The antioxidant MitoQ was more successful at decreasing ROS burden than BAY-82, with reductions of 19% and 56% at 6 and 24 hpi, respectively. In each case, the therapeutics decreased the ROS burden in TC-83 infected cells below the ROS burden experienced in uninfected BV2 microglia. We predicted that a decrease in overall ROS burden would correspond with an increase in MMP. Our data indicates that BAY-82 and MitoQ treatment not only recovered MMP in infected cells, but that membrane potential was higher in the treated samples than in the uninfected mock control (Figure 6e). At their most effective, BAY-82 and MitoQ increased membrane potential by 29% and 48% over the 4 hpi solvent control. These results indicate that the mitochondrially directed antioxidant is more effective at restoring mitochondrial function than the anti-inflammatory BAY-82.

### 3.6. Role of Mitochondrial Oxidative Stress in TC-83 Induced Inflammation

#### 3.6.1. Mitochondrial Dysfunction Contributes to Direct Inflammatory Burden

**Figure 7:**
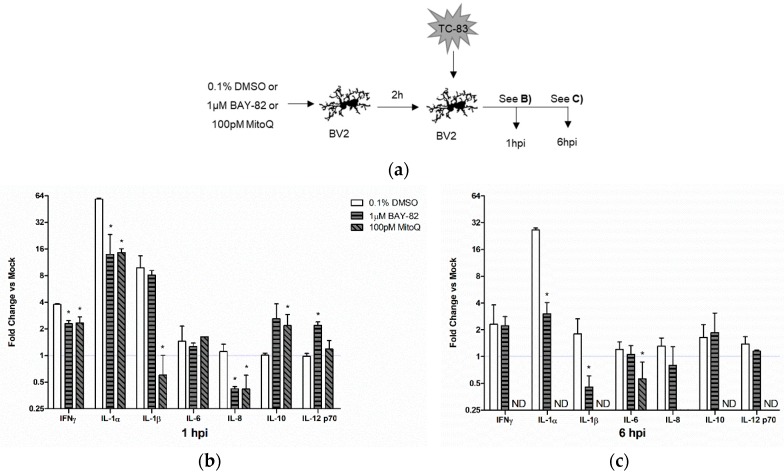
Mitochondrial oxidative stress contributes to direct TC-83 induced inflammatory burden. (**a**) Experimental workflow indicating BV2 microglia were pre-treated with either BAY-82, MitoQ, or the DMSO solvent control prior to inoculation with TC-83 (MOI:2). Inflammatory cytokines produced by BV2 microglia as the direct result of TC-83 infection were measured at (**b**) 1 h and (**c**) 6 h post infection. The quantitative data are depicted as the means of three biologically independent experiments ± SD. * indicates significant difference (*p* < 0.05) between the indicated sample and the time-matched DMSO control.

We wanted to determine how mitochondrial stress in TC-83 infected microglia might be contributing to the inflammatory cytokines released by these direct infected cells. To this end, we pre-treated BV2 microglia with either BAY-82, MitoQ, or the solvent control (DMSO) prior to infection with TC-83 (Figure 7a). At 1 hpi, both BAY-82 and MitoQ treatment had significantly reduced levels of IFN-γ, IL-1α, and IL-8, in addition to significantly increasing the levels of the anti-inflammatory cytokine IL-10 (Figure 7b). Interestingly, only MitoQ was able to significantly decrease levels of IL-1β, indicating this cytokine might be directly induced as the result of oxidative stress in TC-83 infected cells. At 6 hpi, BAY-82 only reduced levels of IL-1α and IL-1β when compared to the solvent control (Figure 7c). Conversely, treatment with MitoQ decreased cytokine levels below the limit of detection for all cytokines queried, except IL-6 and IL-8 which had decreases of 44% and 98%, respectively when compared to the solvent control. These results support the hypothesis that mitochondrial oxidative stress contribute to the inflammatory response in TC-83 infected BV2 microglia.

#### 3.6.2. Mitochondrial Dysfunction Contributes to Indirect Inflammatory Burden

**Figure 8:**
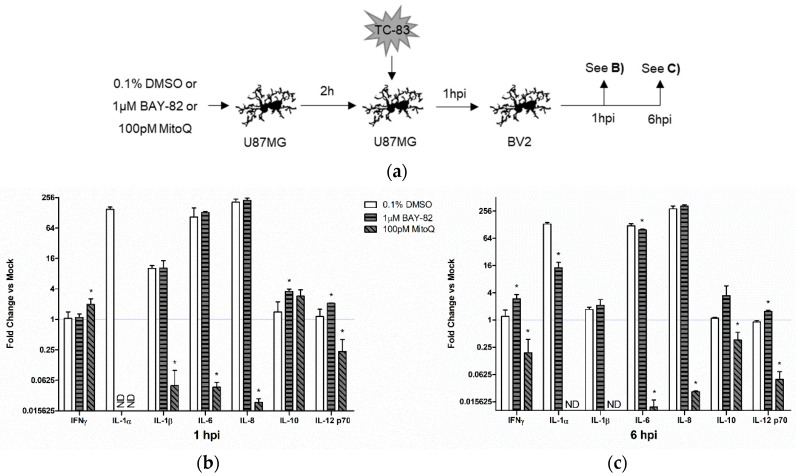
Mitochondrial oxidative stress contributes to bystander induced inflammatory response. (**a**) Experimental workflow indicating U-87 MG astrocytes were pre-treated with either BAY-82, MitoQ, or the DMSO solvent control prior to inoculation with TC-83 (MOI:2). Supernatants were removed from infected astrocytes at 1 hpi and overlaid onto BV2 microglia, with inflammatory cytokine production being measured at (**b**) 1 h and (**c**) 6 h post overlay. The quantitative data are depicted as the means of three biologically independent experiments ± SD. * indicates significant difference (*p* < 0.05) between the indicated sample and the time-matched DMSO control.

While controlling the direct inflammatory burden is important to improve disease outcomes, it is paramount that bystander inflammation be controlled given its overall contribution to the inflammatory microenvironment. To determine the involvement of mitochondrial oxidative stress in perpetuating bystander inflammation, we pre-treated U-87 MG astrocytes with BAY-82, MitoQ, or the solvent control prior to infection with TC-83 (MOI:2). At 1 hpi, we removed the supernatants from the infected astrocytes and overlaid them onto naïve BV2 microglia (Figure 8a). We then queried cytokine levels at 1 and 6 h post overlay. Treatment with the anti-inflammatory BAY-82 resulted in the increase of cytokine production at 1-h post overlay (Figure 8b) and 6 h post overlay (Figure 8c). The only notable decrease with BAY-82 treatment was a 23-fold reduction in IL-6 at 6 hpi. Conversely, treatment with MitoQ led to significant decreases in numerous cytokines, including IL-1β, IL-6, IL-8, and IL-12p70. While initially increased at 1-h post overlay, IFN-γ was also significantly reduced with MitoQ treatment at 6 hpi when compared with the TC-83 infected solvent control. The contrasting cytokine profiles elicited by microglia cells exposed to BAY-82 and MitoQ treated astrocytes indicates that mitochondrial oxidative stress more greatly contributes to the overall inflammatory microenvironment than general anti-viral inflammatory responses. These results also indicate that controlling oxidative stress with a therapeutic such as MitoQ could improve disease outcomes by significantly reducing overall inflammatory burden.

## 4. Discussion

Viral infection of neuronal tissue is often associated with a significant inflammatory response resulting in damage to the blood brain barrier (BBB). Disrupting BBB integrity has been linked to long-term neurological consequences, and therefore it is important to consider the collateral damage that can be elicited by bystander microglial cells that contribute more significantly to the overall inflammatory load than the direct infection itself. In these cases of neuroinflammation, effective therapeutic interventions must target the virus and the inflammatory events to protect the host from long-term damage.

In the pursuit of a murine model of VEEV-induced neuroinflammation, we first had to characterize how VEEV would interact with murine microglia. We utilized the TC-83 strain of VEEV because it is well documented to induce robust inflammation during the early stages of disease progression in the brain [44,46,47]. Our data indicates that TC-83 effectively replicates in BV2 murine microglia, with infectious viral titers, rates of viral genomic replication, and induction of the caspase response similar to that of the previously established U-87 MG astrocytoma model (Figure 1).

Our previous work investigated the role of mitochondrial dysfunction in perpetuating TC-83 infection of human astrocytes. Given the similarities in viral kinetics, we predicted that murine microglia would undergo similar levels of mitochondrial dysfunction when infected with TC-83. Our results indicate that microglia undergo mitochondrial stress, with accumulated reactive oxygen and decreased mitochondrial membrane potential during infection (Figure 2). However, we noted that the extent of ROS accumulation was less than that of the U-87 MG model. We attribute this difference to microglial dependence on ROS species, particularly oxidant production by NADPH oxidase, for proper immune function [48]. Microglia employ more antioxidant systems than astrocytes as the result of this ROS-dependence, which could explain the decreased accumulation of ROS in TC-83 infected BV2 microglia when compared with the U-87 astrocyte model.

Microglia activation is driven by a variety of neurotoxic molecules, including lipopolysaccharide, IL-1β, IL-12, and double-stranded RNA [49]. These stimulation events lead to a redox sensitive upregulation of CD11b in activated microglia, whereby ROS will upregulated CD11b expression via nitric oxide [49,50]. We confirmed that BV2 microglia are activated during TC-83 infection using time-lapse microscopy to measure morphology changes and flow cytometry to measure CD11b expression (Figure 3). Confocal analysis of the mitochondrial networks in infected microglia also confirmed the change in morphology, which corresponded to a perinuclear clustering of the mitochondrial networks. This phenotype, while typically indicative of mitochondrial damage, is partially attributed to the morphology changes that activated microglia undergo.

Microglia are already implicated in amplifying many virus-induced neuropathologies [42,51,52,53,54]. Thus, we predicted that the high level of interconnectivity between microglia and other CNS components makes it highly likely that the inflammatory responses elicited by microglia would contribute to disease progression. Our analysis indicates that direct TC-83 infection of microglia results in production of numerous pro-inflammatory cytokines, including IFNγ, IL-1α, IL-1β, and IL12p70 (Figure 4). While this cytokine induction certainly contributes to the overall pro-inflammatory state, we suspect that the transcriptional inhibition activity of VEEV capsid could be inhibiting transcription of pro-inflammatory cytokines in directly infected cells. Thus, in order to properly characterize the effects of TC-83 elicited CNS inflammation, we wanted to determine to what extent infected cells could elicit microglial pro-inflammatory actions. Our results indicate that these bystander microglia have a significantly greater induction of the key microglia pro-inflammatory cytokines, including IL-1β, IL-6, and IL-8 (Figure 5). This cytokine induction happens within 1 h of infection, corresponding with the microglial activation profile described in Figure 3, and lasts for at least one round of viral replication (6 h). The rapid induction of bystander inflammation, and the extent to which this pro-inflammatory state is established, could have significant impact on overall disease progression and may contribute to BBB permeability during infection.

Many therapeutic interventions for viral infections target the pathogen’s ability to replicate in host cells. While anti-viral therapies are an important treatment option, we believe that this approach fails to address the bystander induced inflammation, which could still induce deleterious CNS effects even in the presence of therapeutics reducing viral replication. In order to specifically target the ROS specific activation of microglia, we utilized a mitochondrially targeted antioxidant (mitoquinone mesylate) to reduce mitochondrial stress in the context of TC-83 infection. We also employed a well-established NF-κB inhibitor and anti-inflammatory (BAY 11-7082) to elucidate the specific role of mitochondrial ROS in the context of microglial induced inflammation. Pre-treatment of microglia with these compounds resulted in decreased caspase activation, decreased ROS accumulation, and increased MMP (Figure 6). While both compounds had improved cellular outcomes in relation to the solvent control, the antioxidant therapeutic had the most significant recovery, indicating that mitochondrially derived ROS contributes more significantly to these infection phenotypes than the NF-κB inflammatory response. The decreased mitochondrial stress also corresponded with a decrease in TC-83 induced inflammation in direct infected microglia (Figure 7). While the anti-inflammatory BAY 11-7082 decreased a number of pro-inflammatory cytokines, those receiving the mitochondrial antioxidant mitoquinone mesylate had significantly fewer cytokines produced during infection. More importantly, this decrease also applied to cytokines induced by the bystander microglia (Figure 8). Here, the broad anti-inflammatory reduced a few cytokines and led to a modest increase in the anti-inflammatory IL-10, but only the antioxidant therapy dramatically reduced cytokine production of the key inflammatory regulators IL-1β, IL-6, and IL-8. This indicates that an antioxidant therapy targeting mitochondrial stress could significantly improve disease outcomes by dramatically reducing the overall inflammatory burden in the CNS.

This study contributes to the overall understanding of VEEV-induced inflammation in the CNS, further elucidating the role of mitochondrial stress in perpetuating deleterious neurological outcomes. Our findings will aid in the establishment of more accurate in vitro and in vivo murine model of infection, which acknowledges the mitochondrial contribution to CNS and BBB damage in the context of VEEV-infection. Ongoing studies will elucidate how these observations replicate in vivo, and in the context of the fully virulent strain of VEEV.

## Figures and Tables

**Figure A1:**
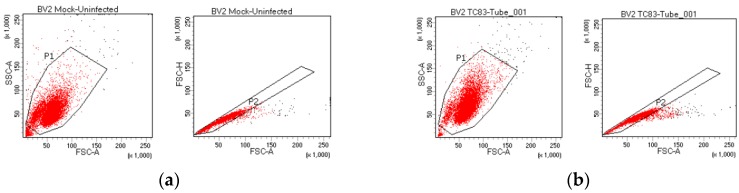
Gating strategy for Figure 3d. (**a**) Uninfected and (**b**) TC-83 infected (MOI:2) BV2 microglia and were collected at 2 hpi and fixed using paraformaldehyde. Cells were stained with phycoerythrin-conjugated Iba1 (PE-A) as a positive control for selection of microglial cells. A phycoerythrin-cyanin5-conjugated CD11b (PE-Cy5-A) antibody was used to determine microglial activation in the context of TC-83 infection. Unstained cells were maintained as a negative control for fluorescence.
